# Byways of Medical Relief
*Previous articles of this series have appeared in The Hospital of October 7 and 21, November 18, December 2, January 6, February 10 and 17, and March 2.


**Published:** 1912-03-16

**Authors:** 


### BYWAYS OF MEDICAL RELIEF.*

(Contributions are invited to this column.)

### IX.—Little Homes for the Blind.

In 1901 the blind numbered 25,317 in England and Wales, 3,253 in Scotland, and 4,253 in Ireland. Of these it is a melancholy fact that 40 per cent. were inmates of workhouses or Poor Law Infirmaries.

The blind are differentiated from every other class of infirm persons by their need of a special environment. Their condition appeals to the most thoughtless, and many admirable charities have been formed with a view to alleviating their lot. There is grave reason to doubt the expedience of removing blind people to institutions.

### How Workhouses Fail with the Blind.

Many who have had experience of the blind believe that they suffer least from the deprivation of sight when they make part of ordinary family life, where their infirmity calls out continual kindness of a spontaneous character, and where their faculties are exerted to the utmost to keep pace with those who see. It would, we think, be injudicious to remove any blind person from a situation, even of some hardship, in which these natural advantages could be enjoyed. But there are blind persons who have no relatives, no home, no aptitude for making friends, no occupation.

There can be no doubt that the blind are liable to suffer in many ways when housed in workhouses. The spirit of altruism does not flourish very happily among the inmates of these establishments, and the sense of degradation which accompanies the normal member of society to the workhouse wards is apt to be intensified in persons who get little relief from their own thoughts, and are often exceptionally sensitive through the quickening of their other faculties. Hence there is a strong motive for providing the blind whose little income will not suffice for independence with homes where their infirmities are considered and their long hours are lightened with suitable occupations. In days to come, when at length the mixed workhouse shall have faded into oblivion, it will be found possible to group together the indigent blind of every county or district in homes where their misfortune is taken into count and skilfully alleviated. For the moment it is to the little private homes we must look for this merciful piece of work.

### What The Blind Need.

It is not altogether easy to establish ideal homes for the blind. To do it well costs more money in proportion to the cases received than many other kinds of charitable work. Those who contemplate undertaking it, or are attempting to do so, should make themselves acquainted with the methods of such wise and progressive institutions as the Normal School for the Blind at Norwood, and with some of the practical work carried on by the County Council Schools for the Blind. The aim should be no mere retreat or refuge for the afflicted, but an opening up of possibilities, a throwing wide of closed doors, even to persons who have long acquiesced in a maimed existence. A situation should be chosen which will admit of the blind taking exercise without an attendant. This is advisable not merely because of the convenience of the management and the expense of providing guides, but also for the mental health of the inmates, who gain much by the effort of walking alone and the habit of moving about freely in the open air. When large grounds are unobtainable, an effort should be made to get access to quiet lanes or open ground where, with practice, independent walks become entirely safe. The Home Teaching Society for the Blind (53 Victoria Street, S.W.), who send instructors all over the country, would no doubt co-operate in teaching Braille. Other occupations also, some of which could be remunerative, should be attempted. The physical side of the malady should not be neglected. Operations rejected with horror after long solitary brooding on the matter will be faced, perhaps, with cheerful acquiescence when body and mind have been braced by good food and the society of sensible people. A little hope, a little courage, and the over-excited nerves recover their balance, and a cure may result.

### Homes Doing Good Work.

Homes for the temporary reception of the blind, where they can have change, good cheer, and instruction, are greatly needed in connection with all big towns. We append a list of some institutions devoted to the blind, a few of which receive them permanently and others for a short term.

At Clifton there is a home for thirteen blind women; at Cheltenham a home with eight beds for the blind of the upper classes; Rockliffe Home for Blind Women at Hull contains ten beds; the Cottage Home for the Blind has a house at Leicester with four beds for women, and at Wycliffe accommodation for twenty persons; the Aged Christian Blind Society has separate homes in North London for men and women and a seaside cottage at Southend; the Manchester Home for Blind Women has twelve beds; the St. Leonard's Seaside Convalescent and Holiday Home for the Blind has separate houses for men and women; at Scarborough there is an industrial home for the blind; at Southport there are homes for fourteen men and fourteen women in connection with the Godfrey Ermen Memorial.

Much more needs to be done, and it will be seen from the statistics given above that these good enterprises do but touch the fringe of destitution to which the majority of blind persons are reduced by reason of their infirmity.

* Previous articles of this series have appeared in The Hospital of October 7 and 21, November 18, December 2, January 6, February 10 and 17, and March 2.

